# Meaning Making in a Retirement Migrant Community: Religion, Spirituality, and Social Practices of Daily Lives

**DOI:** 10.3389/fpsyg.2021.707060

**Published:** 2021-08-18

**Authors:** Jenni Spännäri, Hanne Laceulle

**Affiliations:** ^1^School of Theology, Faculty of Philosophy, University of Eastern Finland, Joensuu, Finland; ^2^Humanism and Social Resilience, University of Humanistic Studies, Utrecht, Netherlands

**Keywords:** meaning in life, aging, retirement migration, religion and spirituality, authenticity, spiritual seeking, baby boom aging

## Abstract

Meaning in life has also been seen as crucial to well-being, and especially, in later life. This study focused on the social complexity of meaning making processes and the role of religion and spirituality in them, by finding out the following: (1) How are meaning-making practices connected with religion and spirituality for Finnish retirement migrants of the boomer generation? (2) What does the role of religion and spirituality in meaning-making practices teach us about the relationship between individual and social aspects of meaning making? This was done by examining a particular group of older persons: Finnish retirement migrants aged 60 or over in Costa del Sol, Spain. The material for this study consists of 58 texts (written correspondence, dataset 1, year 2009), 10 semi-structured interviews (dataset 2, year 2011), and 30 completed online surveys with open-ended questions (dataset 3, year 2019). Key findings include that religion and spirituality are present in the lives of our informants in a variety of ways, playing a significant role in their meaning making, and that they appear as intertwined and not so easy to separate. A variety of religious and non-religious forms of spirituality exist in this population, and all of these forms can be relevant factors in meaning making. Also, the engagement in meaning making, contrary to what has been suggested in some of the literature about meaning in later life, not only occurs in response to confrontations with health issues, death, or other major life events. Instead, we found that meaning making occurs as a process that is often inherent to daily activities which may seem “trivial,” but in fact turn out to be important sources of purpose, values, and connectedness. Contrary to the dominant modern ideal of the authentic, self-sufficient human agent, which is based on a problematically atomistic and individualistic anthropology, for our respondents, their authentic subject position is embedded in the social practices of their daily lives, which nourish their individual spirituality and are vital to making meaning.

## Introduction

Meaning in life has also been seen as crucial to well-being, and especially in later life, manifested for example, in the need for life review and adjustment to changing roles in the family and society (Reker et al., 1987; Krause, 2012; Crescioni and Baumeister, 2013; Krok, 2014). In this article, we focus on the role of religion and spirituality in the process of meaning making in later life. Although viewed as central, the contributions of religion and spirituality to meaning making have not been examined in research adequately or in their full spectrum.

The role of religion and spirituality for meaning making in later life is particularly salient for the postwar baby boom generation, a generation differing from its predecessors in a significant way (Roof, 1993, 1999; Pruchno, 2012). Although the boomer generation has been shown to either stay quite stable in their religious orientation and its intensity, or to become more religious by age and in various existential challenges (Silverstein and Bengtson, 2018), it is also the first generation whose bonds with traditional religious institutions started to loosen (Roof, 1993, 1999). At the same time, this generation developed a profound interest in spiritual practices advancing personal growth and development, often leading to complex trajectories of spiritual seeking (Atchley, 2009). As a result, many older people nowadays no longer feel (completely) at home with traditional religious institutions and their teachings and practice, and are searching for their own spirituality. Often, they are creatively using sources from different religious traditions to fulfill their need for meaning, a profile that can be characterized as “hybrid religiosity” (Berghuijs, 2017).

Influenced by the broader societal transition toward a culture of authenticity and personal fulfillment (Taylor, 1991), the relationship between spiritual authority and religious institutions has become self-chosen rather than inherited or non-chosen for the boomer generation and subsequent cohorts. As a result, taking an active role in identity building and meaning making in the global framework of religion and spirituality, facing various types of religious and non-religious convictions, has increasingly become a necessity (Flory and Miller, 2008). Members of the boomer generation characteristically rely more on their own judgement, feelings, and intuition regarding their beliefs, what to accept and what to reject, than on, e.g., traditions or religious authority (Coleman and Mills, 2019, p. 104, 105). At the same time, the social and communal settings of religion and spirituality remain profoundly important in meaning making (Silberman, 2003).

We may conclude that the boomer generation is exemplary for a generation immersed in the religious and spiritual transitions characteristic of a secular age (Taylor, 2007). Its diversity in religious and spiritual orientations makes this generation particularly interesting for studying the role of religion and (religious or non-religious) forms of spirituality in meaning-making practices. In this article, we will look specifically at how their meaning making practices unfold in an interplay of personal and social orientations toward religion and spirituality and how these meaning-making practices are connected with religion and spirituality.

We study these themes in a specific social context, namely, a Finnish retirement migrant community in the Costa del Sol, Spain. The specific characteristics of this population provides us with an opportunity to study the interaction between individual and social aspects of meaning making in the light of religion and spirituality, because, as we will discuss below, both communal bonds and personal searching motives are strongly present in this community, as well as the stimulus for meaning making in the transmigrant context.

Our study thus aims to increase the understanding of meaning in life as an influential well-being factor of the current and future generations of older persons, and to shed light on the social complexity of meaning-making processes in relation to the role of religion and spirituality for these people. To that end, we will answer the following research questions:

1. How are meaning-making practices connected with religion and spirituality for Finnish retirement migrants of the boomer generation?
2. What does the role of religion and spirituality in meaning-making practices teach us about the relation between individual and social aspects of meaning making?

## Background

### Meaning and Later Life

Research on meaning and meaning-making is an exciting and growing field that has been approached from different disciplinary angles, including theological, philosophical, and psychological studies (Hupkens et al., 2018; Schnell, 2021). Partially because of this disciplinary variety, however, consensus on how to define meaning is still lacking, though an integrated approach is generally agreed to be desirable (George and Park, 2017). Whether meaning is defined in terms of a set of psychological or psycho-social needs, a global system of beliefs and values or rather a set of situated practices, of course impacts methodological choices in the study of the phenomenon of meaning as well.

In the literature, there exists many distinct ways of defining meaning. These include Wong's model, which defines global meaning in life to consist of the following four components: purpose, understanding, responsibility, and emotion/enjoyment (Wong, 2014); Steger's approach which distinguishes among purpose, significance, and coherence (Baumeister, 1991; Martela and Steger, 2016) defines the following needs for meaning: purpose, efficacy, moral worth, and self-worth, to which Derkx et al. (2019) added needs for coherence, comprehensibility, and excitement; Park (2010), who distinguishes between global and situational meaning; or Edmondson (2015) who takes an ethnographic approach and presents meaning not as answering a predefined set of needs, but rather as an emergent phenomenon created in the daily life practices of people.

Meaning-making practices are often linked to frameworks of meaning, also called meaning systems (Taylor, 1989; Eidelson and Eidelson, 2003; Silberman, 2003, 2005). They are to be distinguished from the scientific conceptualization of meaning listed above; rather, these are frameworks held by individuals who serve as background of their understandings of themselves and the world. Meaning systems are theories and beliefs about both self and the surrounding realities, used in decision-making, planning, defining the internal and external relationships, and attitudes of an individual, in general, making sense and giving meaning to the experiences of an individual. Meaning systems are contextual, both in relation to the social and historical settings where experiences happen, and in relation to the personal history and life course of an individual (Taylor, 1989; Silberman, 2003, 2005; Fischer, 2009; Uwland-Sikkema et al., 2018). This conceptualization of meaning system comes closer to several other concepts, including “horizons of interpretation” (Heidegger, 1927/1962; Hirsh, 2013), in the way it refers to guiding the meaning-making processes of individuals.

On the one hand, meaning systems are personally and individually appropriated, but on the other hand, they are always at least partially shared with others—persons, communities, and larger social entities like societies. Shared meaning systems contribute to the shaping and preserving shared realities in a group, and to create a shared sense of communality and belonging (Bar-Tal, 2000). By using shared meaning systems, the communities and their members are able to direct their behavior and interpretation of events, including historical ones (Kelman, 1997; Hirsh, 2013). The shared realities shaped by meaning systems not only affect the everyday life of individuals and communities, but also more abstract issues and experiences, including views on what is meaningful in life and where meaningful experiences can be searched for (Silberman, 2005).

Religions are examples of social phenomena which can be approached as global meaning systems. As they most often explicitly deal with questions related to the sacred and the transcendent, they can constitute an influential framework not only on meaning and meaning making, relating to beliefs, but also on contingencies, expectations, and goals (Lewis Hall and Hill, 2019). However, it is vital to note that religions are by no means the only possible frameworks for a meaning system. Also explicitly non-religious, as well as spiritual but not religious, meaning systems are found in diverse social groups, including the baby boomer generation (Pargament, 1997, p. 32; Silberman, 2003; Pargament et al., 2005).

George and Park (2017) suggests that global meaning systems always encompass dimensions of cognition (beliefs people hold on how the world is), motivation (goals that people feel motivated to pursue in their lives), and emotion (the subjective sense of meaning being present or lacking in life). With regard to situational meaning, Park's model distinguishes between appraisals of a certain situation in terms of its meaning, comparison of this appraisal with the *global meaning system* (and possible distress if the given situation is not concurrent with it), *meaning making*, which describes the efforts people make to create or restore coherence between the global meaning system and a given situation, and *meanings made* (the resulting adaptations in the global meaning system).

Aging and old age can pose specific challenges when it comes to meaning making. First of all, as Krause (2012) suggests, the emphasis in meaning making may shift during the life course, so that setting goals may be more important in constituting meaningfulness for younger people, whereas the ability to reconcile the past and look back on the life of an individual with peacefulness and contentment gains in importance during later life. Second, modern Western societies are often hostile toward aging people, because old age is associated with decline and fear, and this cultural hostility impedes the chances of older people having to experience their life phase and social roles as valuable and meaningful (Edmondson, 2015; Laceulle, 2018). On the other hand, the second half of life has also been traditionally seen as a phase in which the human potentials for wisdom and spiritual growth can be fully realized (Tornstam, 2005; Atchley, 2009; Edmondson, 2015).

In the current literature, the function of religion and spirituality for meaning in later life is particularly explored with regard to the confrontation with death, and with regard to coping with health problems. Manning (2019) states that religion can serve two important functions in coming to terms with the existential reality of finitude, one's own death or the death of others. First, religion can provide people with a framework in which events of life can be experienced as ordered and coherent, rather than random or chaotic. This makes it easier to feel a sense of purpose and accept that death is part of the human condition. Second, religions can offer people action steps to deal with death (for example in the form of rituals), that give them a sense of control and reduce possible feelings of helplessness and despair in the face of death.

The role of religion and spirituality in meaning making is also linked to coping with health issues in older age (Krok, 2014; Manuti et al., 2016; Xu, 2016). Although meaning can be searched for and found in both religious and non-religious contexts, there are differences between how different meaning systems propose to deal with health issues, for example, with regard to how they define the ideal emotional state and its path leading (Park and Hale, 2014; Tsai et al., 2016). Studies show that being non-religious need not lead to worse health results compared to being religious, and that atheism or non-religious forms of spirituality and hybrid religiosity are equally functional in providing sources of meaning in diverse life situations, including confrontations with diminishing health and death (Wilkinson and Coleman, 2010; Coleman and Mills, 2019). What remains mainly unstudied is what the role of religion and religious or non-religious spirituality can be in meaning making aside from situations where one is confronted with death or diminishing health.

### Religion and Spirituality

The concepts “religion” and “spirituality” are both fuzzy and overlapping. Where religion often refers to beliefs, behaviors, rituals, and ceremonies related to an established tradition, spirituality evolves more around the theme of transcendence, within or without organized religion (Koenig et al., 2012). In this article, the concepts are useful especially as tools for working with and illustrating the cultural and linguistic richness and differences related to these phenomena (Murphy, 2018). These differences and variations have been observed in various cultural contexts including in Japan (Takahashi, 2020). From longitudinal and narrative data pertaining to the relation of religion, belief, and spirituality in old age, Bengtson and Johnson (2016) drew some interesting conclusions about how the role of religion and spirituality has transformed across different generations during the twentieth and early twenty-first centuries. First, they observed how conceptualizations of God have changed, showing that older cohorts are more likely to see God as a transcendent, omniscient, and distant being, whereas the younger cohorts often see God as an imminent and internalized power to whom they have access in themselves. Second, a comparison between the older and younger generations shows an increasing separation of religious practice from religious institutions. Whereas, for older generations it was self-evident that religion is practiced in church, synagogue, or mosque, the younger generations increasingly came to the conviction that leading a religious life did not necessitate regular church attendance, but could also be realized in other practices outside traditional houses of worship. Third, religion and spirituality have increasingly become two different domains for younger generations, as compared to older cohorts. While religion is increasingly identified with organized, institutionalized traditions, spirituality became the term for an internal, personal relationship with the Divine, to be obtained through processes of spiritual development, growth, and transformation.

The contemporary religious and spiritual landscape is best understood as a complex and multilayered structure. It is typical for the “quest culture” of modern spirituality that traditional boundaries characteristic of religious traditions and institutions no longer suffice to understand the meaning making processes that people engage in (Roof, 1999). Instead, in the process of spiritual seeking, people are shaping their own “lived” practices of religion, freely drawing on sources both inside and outside traditional religious frameworks. As Roof puts it, “Agency, or the role of the individual actively engaging and creating an ongoing personal religious narrative in relation to the symbolic resources available, is (.) crucial to our understanding of contemporary spiritual quests” (Roof, 1999, p. 12). Thus in this article, we put emphasis on meaning making as an active process, in which people may or may not draw on religion and spirituality to create meaning (Manning, 2019).

### Meaning Making and Spiritual Seeking

In this article, we specifically aim to look at meaning *making*, which we broadly define as the active component of the engagement of people with meaning in life. Meaning making not only pertains to global life meaning, but also occurs as people ascribe meaning to specific aspects of their existence. Making meaning can be distinguished from *finding* meaning, which implies a more passive role for the individual. Also, the verb “making” emphasizes the active construction of meaning by individuals in their lifeworld, whereas “finding” assumes that meaning has an existence independent of individual consciousness in the outside world. Meaning making is related but not equivalent to searching for meaning. According to Park (2017), meaning making occurs when a discrepancy is experienced between the global meaning system (beliefs and values) that people adhere to, and to a specific situation. For example, experiencing a traumatic life event, such as the loss of a loved one, may alter the philosophical or religious outlook of an individual on life, and affect the view of the world of an individual and the perception of an individual on himself. The process of meaning making then facilitates the repair of a coherent match between the global and situational meaning (Park and Gutierrez, 2013). However, as ethnographic approaches to meaning in life suggest (Edmondson, 2015; Derkx et al., 2019) meaning making is not necessarily restricted to our response to situations of distress, but can also be approached as a continuing practice throughout our daily life activities. We would like to take this broader perspective to not only focus on the distress that discrepancies with global meaning systems pose, but also on more “ordinary” instances of meaning making as an active process of creation.

Manning (2019) suggests that meaning making processes in which we assign meaning to the past are different from meaning making processes in which we try to find meaning in what happens to us in the present. This is relevant in the context of aging, because evaluating the life of an individual with the purpose of finding what Erikson (1997) has called “ego-integrity” is generally seen as an important meaning-goal in later life. Whereas, Manning conceives the past-oriented type of meaning making as a mainly intellectual exercise where events from our past lives are put in an interpretive narrative framework (which may or may not be religiously inspired), the present-oriented type of meaning making is less about reflection and explanation to create a retrospective coherence, and more about action, emotion, and motivation to create a sense of purpose and control amidst the events one lives through at that moment.

The reflective and retrospective orientation characteristic of meaning making about the past is consistent with the philosophy that people are narrating beings, whose meaning making relies heavily on narrative (Ricoeur, 1992; Roof, 1999; Manning, 2019). People tell narratives to shape their identity and express their relationships with other people and with the values and practices of their societies (McAdams, 1993; Smith and Halligan, 2020). For older persons, narrative seems to gain an increased importance as an instrument of creating meaning. As many authors in the tradition of narrative gerontology underscore, in looking back at the life of an individual and construing a coherent narrative about it, people come to terms with themselves and, ideally, can perceive their life in retrospect as purposeful and significant (Kenyon et al., 2011; De Medeiros, 2013).

In this article, we relate our focus on meaning making to processes of spiritual seeking. According to Wuthnow (1998), we can observe a transition from religious dwelling, where people feel at home in a particular tradition and community, usually provided by religious institutions, toward spiritual seeking, where people undertake a highly personal journey of transformation and, hopefully, growth in which they aim to discover and create their own meaning-generating spiritual orientation. The process of spiritual seeking can take place in a fruitful dialogue with more traditional religious institutions, such as church communities, but can also involve distancing oneself from them and creating new forms of communion with like-minded spiritual seekers. Though the study of Wuthnow focuses on the North American context, the transition from religious dwelling to spiritual seeking can be found throughout the entire Western modern world.

The shift from religious dwelling to spiritual seeking comes with a strong focus on inwardness, subjectivity, and authentic personal experience as the focal point of religious and spiritual energy (Roof, 1999). This is exemplary of the modern turn toward an ethics of authenticity described by several authors (Taylor, 1991; Ferrara, 1998; Guignon, 2002; Laceulle, 2017). Taylor (1989) has provided an eloquent historical-philosophical analysis of this shift toward an ethics of authenticity. The study of Taylor sketches the genealogy of the meaning systems relied upon by people in Western modernity, as they relate to their self-understanding and identity. The search for meaning has evolved into an inner journey that is perceived as highly individual and authentic. A problematic side of this discourse is that it seems to present a rather atomistic image of the human agent, with hardly any sensitivity for how agency is embedded in social practices. Rather, social influence is presented as a threat to living authentically (Laceulle, 2017). However, as Taylor pointed out, it would be a mistake to see the pursuit of authenticity as a purely personal process; instead, it is deeply intertwined with the moral horizon of the culture/society in which it takes place and therefore always socially mediated. According to Taylor, it is impossible to develop an individual vision of “the good” (indispensable for experiencing meaningfulness) without knowing where you stand in a field of socially constituted “goods” that form the moral horizon of the culture of an individual. We can learn from the analysis of Taylor that the spiritual seekers of our research population may have an ambiguous relationship with traditional contexts of religious dwelling, but that their search or quest for meaning is nevertheless a deeply social process.

It would therefore be a simplification to state that spiritual seeking, as opposed to religious dwelling, is predominantly an individual process, and that people no longer feel themselves to be part of traditional religious and social communities, with potentially detrimental consequences for their sense of belonging and for social cohesion in society. Instead, the population of spiritual seekers experience “multiple religious belonging,” though no longer identifying as exclusive members of a certain religious institution, people can still “feel at home” and experience a sense of authentic relation with multiple religious sources and traditions (Berghuijs, 2017). Traditional boundaries between religious and spiritual traditions have become irrelevant to some extent, but the sources of meaning they provide gain significance in new creative combinations.

## Materials and Methods

This article examines these questions within a particular group of older persons: Finnish retirement migrants in Costa del Sol, Spain. This group is characterized by constant change and fluctuation: the typical lifestyle includes spending summers in Finland and winters in Spain, members of the community come and go, and a large proportion of social activities and networks are bound to time and place: either Finland or Spain (Karisto, 2005, 2008a; Oliver, 2008; Spännäri, 2013). Also, the individuals engaging in this lifestyle experience transitions: from work to retirement, from a country to another, from a social or family role to another. Many of these older migrants are best described as transmigrants (Glick Schiller et al., 1995; Gingrich and Preibisch, 2010), sharing their time and interests in varied proportions between Finland and Spain. This is one of the reasons why this particular population is an intriguing context for studying meaning making. Retirement migration and transmigration, involve not only various transitions and challenges which require self-realization, personal coping, and social relating, but also adaptation to new things and coping with loss—all of which are circumstances where meaning making is especially needed (O'Reilly, 2000; Gingrich and Preibisch, 2010; Warnes, 2010; Spännäri, 2013).

Another reason for focusing on this particular, Finnish group of older persons, are the country-specific characteristics of the baby boomer generation in Finland. Both the size of the baby boomer generation and its timing and span are exceptional, when compared to other countries. The exceptionally large After-War birth cohorts were proportionally larger and their birth years fewer than in most other countries after the Second World War. For example, the annual birth rate curve almost doubled in Finland during the second half of the 1940s, whereas in Great Britain, the annual growth was only about 25% (Nieminen, 2007; Karisto, 2008b). Thus, in Finland the concept of baby boomers, as a sociological phenomenon, may have a greater explanatory power than in several other countries and the shift from previous behavior patterns to those adopted by the boomer generations might be more dramatic (Karisto, 2008b). Related to meaning making behavior, a significant shift has been observed in several European and North American countries (Roof, 1993, 1999,). In Finland, where the prevailing narrative has been of cultural and ethnic homogeneity (Keskinen et al., 2019), the choice contexts for meaning making have been relatively limited. Thus, for many older Finns, especially those belonging to generations preceding the baby boomer generation, the search for meaning in life has taken place in a religious framework (e.g., Niemelä, 2011).

Here, arises a third reason for focusing on this community of older persons: according to earlier studies, the retirement migrant community is much more active in religious and spiritual activities than the corresponding demographic groups would be in Finland. In Finland, religious attendance is at the North European low level. In the year 2019, about 68.7% of Finns were members of the Lutheran church, but only 6% participated in a service at least once a month (Salomäki et al., 2020). According to an earlier questionnaire survey, it was found that a Finnish religious service was the second most popular Finnish event at Costa del Sol: about 46% of the informants had attended a service and 29% had attended some other Finnish religious activity during the last winter season (Karisto, 2008a). In a study regarding the Finnish seasonal migrants and regular tourists in Spain and the Canary Islands, it was found that during their stay in Spain, 57% of Finnish migrants attend more to religious services than they do in Finland, whereas only 12% attend more in Finland than in the Canary Islands (Mäkeläinen, 2011). Also, many of the persons attending the services in Spain do not take part in church activities in Finland. In an earlier study, 28% of questionnaire survey informants, who stated that religion is not important to them, still had attended a service during their winter stay in Spain (Karisto, 2008a, p. 248–249). A significantly understudied phenomenon is the emergence of meditation and alternative spiritual practice groups. This can be observed in the social media pages of the community, advertisements in newspapers, and in other traditional media from around 2015 onwards, but has not yet been studied.

The obvious and explicit explanation for the higher attendance in Spain than in Finland is meeting other Finns. Social connections and activities in various associations have been proved crucial to the well-being of retirement migrants and in the creation of social capital (Casado-Diaz, 2009; Simó Noguera et al., 2013). Like many migrant churches and congregations globally, the Finnish religious communities in Spain are important platforms for forming social networks (Ebaugh and Saltzman Chafetz, 2000; Jeppsson Grassman and Taghizadeh Larsson, 2013). However, there are various other instances, contexts, and events outside the religious ones where the Finnish migrants in the Costa del Sol could and do meet each other. While social factors explain some of the attendance, it leaves a particular demand for religious events in hiding.

In a Danish case reported by Warburg (2012), the church was the only or one of the very few available organizers of national and cultural activities and the participation was the highest at the non-religious events organized by the parish. On the contrary, the Finnish community at the Costa del Sol has a variety of clubs, associations, and institutes to organize cultural, educational, and entertaining activities. Also, the weekly services are clearly the most popular activities organized by the Lutheran parish. Religious activities have been strongly associated with the well-being, especially the mental well-being of the migrants, where non-religious activities did not have the same effect (Connor, 2012). The reason might lie in the ability of religion to function as a tool of connectivity both in diasporic and transnational ways (Ebaugh and Saltzman Chafetz, 2000). For these reasons, the retirement migrant community with its abundance of religious and spiritual activities is a very interesting context to study, given our aim to shed light on the social complexity of meaning-making processes in relation to the role of religion and spirituality.

The material for this study was collected in the Finnish community of Costa del Sol during the years 2009–2019. The material consists of 58 texts (dataset 1, year 2009), 10 semi-structured interviews (dataset 2, year 2011), and 30 responses to an online survey with open-ended questions (dataset 3, year 2019). The material is also presented in Table 1: datasets of this study. Together, this combination of narrative material gives a rich, multidimensional view to the life of the retirement migrants and their community across time, and allows for the examination of their meaning making, from various points of view.

**Table 1:** Datasets of this study.

	**Dataset 1**	**Dataset 2**	**Dataset 3**
Year of collection	2009	2011	2019
Format	Texts (written correspondence)	Semi-structured interviews	Survey (open-ended questions)
Number (included in the analysis)	58	10	30

The texts for the dataset 1 were collected by letters and emails, with the cue “Write about religion in the Finnish community at Costa del Sol.” The invitation to write was published in the Finnish language magazines and newspapers in the area, and also advertised through the biggest Finnish religious communities: the Evangelical–Lutheran parish and the Tourist Church. This collection resulted in 64 texts, approximately half a page long, the shortest 10 sentences, and the longest two and a half pages. The informants represented various religious affiliations (which included: Lutherans, Pentecostals, and witnesses of Jehovah), and age groups (from 40 to 84). In this study, we excluded six texts where the informant was known to be under 60 years old, as we focus here on the meaning making, especially in the later life. Thus, 58 texts (40 women and 18 men respondents) constitute the dataset 1 and were used in our analysis. From the texts, we picked the themes and approaches for the interviews constituting the dataset 2 (See interview questions in Appendix A). Eight of the interviewees in the dataset 2 had also provided a text to the dataset 1. The remaining two interviewees were recruited in the Finnish events in the area, and were persons especially willing to contribute to research on religion and spirituality in the area. The interviewees of dataset 2 were aged from 60 to 82, where half of them were men and half women, and they represented Lutheran, Pentecostal, and non-religious orientations. The interviews were carried out by the first author of this article, mostly in the homes of the respondents, and they lasted from 1.5 to 3 h.

Dataset 3 was created by a survey, distributed online in March 2019 to a Facebook group of Finns in Costa del Sol (with 250,000 members), and sent by email to persons affiliated with a meditation/non-religious spirituality group. The survey material provides an interesting incremental point of view to the community, reaching out to specifically spiritual but not religious persons, and the emergence of new spiritual but not religious activities in the community. The survey was anonymous so there is no means of telling whether the respondents have also contributed to the first two datasets. This study examines the replies to open-ended questions included in the survey (Included in Appendix B), which focused on discussing and reflecting on meaning in life, as well as religious/spiritual orientation and practices. We received altogether 65 replies to the survey, of which 30 (8 men and 22 women) were from informants aged from 60 or over, and thus are included in this study focusing especially on meaning making in later life.

The material was analyzed with qualitative content analysis and adapted a grounded theory methodology, using the Atlas.ti analysis software (Charmaz, 2006; Friese, 2011). The first author analyzed the first two datasets, and the third dataset was analyzed by the two authors together, first taking turns and then each author checking on the analysis of the other. All categories and codes used were extracted from the material, then grouped and regrouped to form themes as responses to our research questions (Dey, 2007).

One significant decision made in the analysis and writing process for this article was the question of translating the terms related to religion and spirituality. Both the phenomena and the vocabulary around religion and spirituality evade clear-cut definitions and are context-specific to a large extent (Moberg, 2009; Murphy, 2018). In the Finnish context, in everyday language, the field of religiosity and spirituality is often described using three words: *uskonnollisuus, hengellisyys*, and *henkisyys*. The word, *uskonnollisuus* would refer to religiosity, through dimensions, such as attendance, practice, and dogmatic beliefs related to institutionalized religion, such as a church—thus, we decided to translate that as religiosity. The word, *hengellisyys* would refer to a type of spirituality relating to institutionalized religion, but in contrast with religiosity or *uskonnollisuus, hengellisyys* focuses more on the personal practice and experiences, such as praying, singing hymns, and having a religious worldview. In this article, this is translated as a religious spirituality. The word, *henkisyys*, then, would refer to spirituality, characterized by experiences and personal practice not connected to institutionalized religions. Interestingly, this term could also include phenomena in the fringes between the spiritual and the secular factors, such as philosophical interest or personal growth.

## Results

### Meaning Making in the Retirement Migrant Community

In our material, meaning making was displayed in a wide variety of ways. For some informants, meaning making was quite comprehensive, touching not only the person in question, but persons in general, and also even larger entities like the cosmos or God:

*I believe (i.e., trust and hope) in the ability of a person to perceive his or her modest part of the earth and therefore to work for himself and his immediate surroundings, both mentally and physically. This can be God in us (D3, 33)*.

For some informants, accounts of meaning making are centered around “the little things in life,” such as volunteering:

*But volunteering, it has been the spice of life.—It is very pleasant to be around people, in everyday life. And at the same time feel united with all the parish members. To feel that you can be of help and to be helped yourself at the same time (D1, 23)*.

For this informant, an important part of volunteering in the parish was to be united with others, and this sense of unity was created through the tasks in everyday life. In addition to the pleasantness of doing together, this activity was given the meaning of unity with all the parish members—which might, judging from the word, *ykseys* the interviewee uses, be a particular, spiritual kind of unity.

Volunteer activities had a strong spiritual importance for some of the informants. An interviewee from the dataset 2, a female in her 60s, ponders:

—*That this is really only temporary. That a time will come, when I don't have to worry or take care of anything*.

*(Interviewer: To know, that everything will end [well in any case)*

*Yes, exactly.] And then you can rejoice and rest and just enjoy. And that gives me strength to strive for that goal here in the parish and in my own life and toward other people. And for that reason, too, participate however I can, whatever small contribution I can make, to… make that possible for others who don't know God. That's really our task here. We want to take others with us there, too, some with a bigger and some with a smaller contribution*.

*(Interviewer: And everybody, in their own way, can take part)*.

*Mm, yes. Like washing the dishes or sweeping the floor there, that somebody, a seeker, can come here. That we're here for them (D2, 4)*.

This informant makes meaning of her volunteering in cooking and cleaning duties in the context of religion. For her, volunteering was not only keeping up the hope for a better life after death, but also in this life, giving strength in everyday struggles. In addition, she makes meaning for her duties in the religious community as helping others to find God.

Our material also reminds that meaning making happens in a place and time.

*I go to worship and ponder the sermon on my way home and at home. I help others whenever I can. It gives strength and good spirits (D3, 42)*.

The moments of meaning making are not something separate from the everyday life, but take place during the usual activities like walking home or embedded in the everyday deeds of helping others. The informant tells about pondering a sermon, and then continues to write about practice: helping others, which also has benefits for the helper herself. The practice and the sermon come together. A practical meaning is given to the religious message, and a religious meaning is given to the spiritual practice.

In addition to everyday life religious practices, festivities, and customs linked to them are also one of the frequently mentioned contexts of meaning making.

*For us, it is very important to celebrate the religious holidays in the Finnish way. For example Christmas is a very emotional and an important holiday. All the childhood Christmases come to mind, with Christmas matins, sleigh rides etc. We also reminisce about persons who have passed away, parents, siblings, and all relatives, and the message of Christmas (D1, 15)*.

The informant describes how Christmas is given the meaning of looking back but also reaching out to others in the spirit of Christmas. The message of Christmas seems to be one of connectivity, both to the past but also to the present. As for the past, the personal life stories appear in the material as important contexts of meaning making for these older persons.

*I was born into a home where religion and the morals, behavior, and good manners that come with it are not unfamiliar. These give good guidance on the journey of life, to take into account others, and to respect their view of life and conviction, no matter which religious branch they belong to (D1, 19)*.

Interestingly, for this informant, the tolerant and accepting way of life was given meaning through their strong religious upbringing. Morals and good manners were seen in the religious context, and shaping the whole journey of life of the informant.

One of the key findings of our analysis is that meaning making is integrated in the everyday lives of the people, not only in the times of festivities, but also in the quite usual chores and activities, like volunteering or walking home.

### Religion and Spirituality in the Meaning Making

Our analysis showed that both religion and spirituality played a role in the meaning making, both in the religious and non-religious context. In the religious context, one of the social phenomena framing the meaning-making processes is a religious participation, which is described to be more religious and active for Finns in Spain than in Finland. A clear majority of informants in the dataset 1 stated, even if that was not explicitly asked in the call for texts, that religious participation was much more common and the participation style was much more active for the Finns in Costa del Sol, than in Finland. The place and change of place seemed to play an important role for meaning making for many respondents, as sunshine and the palm trees were often mentioned as pleasant elements but at the same time constant reminders of being in a foreign environment.

“*I think that in Spain (Finnish) people are more open and receptive to religion. They are older and the departure from this world is approaching for everybody. But here also those people go to the church, who won't do it in Finland because of bashfulness or other reasons. The songs and hymns are sung at full blast, although everybody isn't always hitting the right key. It also seems that everyone goes to receive communion.” (D1, 61)*.

In addition to the higher age and approaching the end of life, the informants mention openness and receptiveness as central factors for increased engagement and attendance. But in addition to the increased frequency, this and several other informants write about the difference in participation style: more open, more personal, a result of an individual choice and stressing more an active role in participation.

The specific social context and the transitions linked to the life in it seem to play a key role in the increased and intensified religious engagement.

“*The church here is like a mother duck who calls her ducklings under her wings for shelter and protection and nourishes both physically and spiritually, the church and religion is much more important to us here than in our homeland—aren't we here like scattered in the winds of the world.” (D1, 13)*.

Here, the religious context was seen as framing the meaning making in a new, uncertain, and insecure situation. The perceived safety and protection provided by the religious context was very often mentioned in the texts. It was mainly left unclear, which were the threats to safety that religious context was perceived to be protecting from. Building a social network and social capital (weak and strong ties) in a new community might be a factor here. In addition, the sense of safety seemed to be also spiritual, as in the quote above, and for this informant:

“*And often it's because problems caused by a certain homesickness, loneliness or troubles in integration develop to be a curious internal longing and emptiness, where spiritual circles are starting to give meaning to one's life.” (D1, 52)*.

The informant describes how external factors, like moving to a new country and social environment create experiences of longing and loneliness, which in turn create internal needs, longing to be fulfilled—which in turn leads to meaning making in a new context and with a new sense of urgency.

Interplay between personal, individual aspirations, and the social framework was not always easy. Many informants, such as the persons quoted above, also mentioned possible obstacles, on why they themselves or their peers have not engaged in religious activities in the home country. The peer pressure for not participating while in Finland, and the absence of it in Spain, was explicitly mentioned in several texts.

“*Belief is a personal encounter with Lord Jesus. The Coast gives the same possibilities for it as Finland does. Sometimes even better, when relatives, friends and neighbors are not there, “controlling” (D1, 52)*.

The “lower threshold” to engaging in religious and spiritual activities in Spain than in Finland, mentioned explicitly in 10 of the 58 texts in the dataset 1, gives a reason to think that the need for this engagement exists also in the Finnish context and is not only related to transitions, such as moving to another country to retire. Also, it underlines the need for active negotiation between the personal needs for meaning and the social context. The meaning making practices adopted in the later life depend on many factors, neither on the social norms nor the pursuit for authenticity.

However, the search for meaning was not linked exclusively to the specific social context of being a retirement migrant in Spain, but also and very firmly to the life stage of older adulthood and retirement.

*It is as if you could start your life anew now that you're retired, and do what you really want to and feel it's the right thing to do, that's spirituality today (D1, 46)*.

Interestingly, the writer not only placed spiritual practices as the engagement they were longing for, but also vice versa: to “do what you really want to do,” to explore one's own agency, was indeed a form of spirituality.

Religion and spirituality also play a role in making meaning of severe illnesses in later life—and the threat they pose. One of interviewees in dataset 2, a woman in her seventies, tells about a person living with several illnesses; how she is a very positive and joyous person, and how she often discusses the possibility of an illness-ridden elderhood:

*We just always remind each other, that if He, our Lord, puts us through that schooling, I won't be there alone. He's there with us.—So I'm not afraid, no (D2, 3)*.

Interestingly, the religious and spiritual framework is here related to togetherness and connectedness; explicitly not only with God, but also with the person and persons sharing similar views.

Also informants who did not consider themselves as religious, saw spirituality as significant contexts for meaning making.

*I do not believe in God as seen by Christianity. I believe there is something bigger. I believe things tend to arrange themselves and that something is behind that. I also like to think that my dead father is somewhere, but where, I haven't figured it out yet (D3, 9)*.

For this informant, non-religious spirituality helped to make meaning and sense of life and its events. For others, engaging in meaning making processes in a spiritual context was primarily motivated by self-realization or self-development.

*I treat religion and religious spirituality in the same way as exotic flowers. I look and marvel. I understand non-religious spirituality more broadly; I think it is a work and an exercise that a person does to change themselves (D3, 27)*.

This informant saw the self-development motive to be especially linked with the non-religious spirituality—in contrast with religion and religious spirituality. The informant thus described what they found to be a key difference of religious and non-religious spirituality: the other is to be looked at, and the other to be exercised.

Another informant describes how religion and religious spirituality affects her everyday life, especially having found God:

*It's had a great effect. Like when I think how I've ever been able to make decisions before, for example. And I thought I was good at decision-making. Now I don't need to do anything more than just ask: “Heavenly Father, how is it now with this thing here. Do I sell this flat or not.” And I get answered. In all my matters I get answered. In a way or another (D2, 3)*.

Although the context where this informant practices her religion is strongly religious, as she is an active member of one of the religious organizations in the area, the effect in her life described here arises from a personal spiritual practice, praying. Thus, another key finding is that religion and spirituality are not easily segregated from each other in the data, and they are manifested intertwined. Also, for this respondent, asking God for advice seems to be a personal process, not involving any religious professionals or key figures. In this narrative, getting answered involves agency, in asking and defining an answer. Thus, it is vital to inspect meaning making as an activity, in the lived context, following the course of research on aging in context, ethnographical approaches, and in aging and everyday life (Edmondson, 2005; Degnen, 2012).

In this study, further examining is to be included on the role of the older persons as active subjects of the meaning making process.

### Meaning Making as an Active Process

One of our key findings is that meaning making is an active process for these older persons, and it involves a negotiation between authenticity and self-expression on the one hand and the social framework on the other. For some informants this is very explicit:

*This is my own thought interpretation about belief and Deity: we must grow and develop by ourselves. No pastor or preacher is able to do that (D1, 29)*.

After presenting the development of his spiritual thinking, the informant comes to the conclusion that everyone is responsible for his own spiritual development.

Another informant, who is not very active in any religious community, uses the religious context to make meaning of his aspirations to help others—even if he would have to disagree with others:

*Well, I don't know if my Christian conviction is that strong… But it comes to me from early childhood “from my mother's milk,” the defending of the weak. And, I've seen christianity so, that it should focus on exactly defending the weak.—This is how I see the teachings of Jesus, too. Some disagree and say that it is not only about helping the weak. But I think it is. That's the core of everything (D2, 9)*.

Later in the interview, the informant tells that for him, personally, helping others is the primary way of practicing spirituality. It is not only a moral obligation, but also a spiritual act, part of a larger whole. That ethos has especially affected his life after retirement, as he feels he now has more freedom and time to do good to others.

For some informants, the focus of the meaning-making processes was in the self, like for this informant, describing the meaning of religious service to her:

*Hearing the gospel helps revitalize and strengthens me. I don't have to accomplish anything, but I can just rest and be taken care of by God. This is something I've been missing through many years of work, to safely be a child of God. Now that I have time, I can read the Bible and other spiritual literature. Now I have time to pray, either specially to settle for prayer or then pray by myself while doing the daily chores. My wish here in the sun is to get closer to God (D1, 59)*.

Religious context had for this informant an uplifting and caring effect. Retirement also offered the much needed time to explore the spiritual dimensions of life, perhaps earlier subdued by work and the related lack of time to focus on oneself. For this informant, however, the individual meaning-making process is launched in a shared context, having heard the gospel with others.

Also, in non-religiously spiritual contexts, both a social aspect and the emphasis on the individual agency as well as authenticity were present. For some, meaning making was the first and foremost linked with social connectivity:

*(meaning of life is to) live in such a way that my deeds, my presence and my words would enable my loved ones to fulfill the purpose of life they've chosen with joy and confidence in their own abilities (D3, 33)*.

For others, the motivation was linked more clearly to self-development:

*I want to find myself through the wisdom of spirituality. Who am I? (D3, 3)*.

These individual experiences and fulfillments do not appear in vacuum. Many informants relate the individual sense of meaning in a relational context.

*I find it unlikely that living on a large scale has any purpose. Instead, I think it is good for individuals to experience that their lives matter to someone or something. This kind of individual sense of meaning can be found in different things for different people (D3, 50)*.

In fact, quite often individualist and relational motivations were intertwined:

*(meaning of life is to)Become the best person possible, that is, kind, helpful, and spiritual toward all living things (D3, 61)*.

This informant connects personal development with contribution to the lives of others, and even nature. The best possible person is also the best person for others, not only for oneself. Meaning in life is actively made and measured by kindness, helpfulness—in everyday life.

## Discussion

The lives of the Finnish retirement migrants we have studied in this article are vibrant with meaning making. Both the written and told narratives from our informants paint a lively picture of a community whose members actively engage with each other and with themselves to infuse their daily lives with meaning.

An important finding is that the engagement in meaning making, contrary to what has been suggested in some of the literature about meaning in later life, does not only occur in response to confrontations with health issues, death, or other major life events. Instead, we found that meaning making occurs as a process that is often inherent to daily activities which may seem “trivial,” but in fact turn out to be important sources of purpose, values, and connectedness. This has been noted also in previous and recent studies (Hupkens et al., 2021). This speaks in favor of studying meaning from an ethnographic, phenomenological perspective (cf. Edmondson, 2015), and not limiting research on this topic to more dominant quantitative approaches using measurement instruments, such as the Meaning in Life questionnaire (Steger et al., 2006). It also resonates with the emerging literature about lived religion, in the sense that through studying this engagement, it is possible to examine how meaning making is practiced—quite alike how the study of lived religion focuses on the practice of religion (Knibbe and Kupari, 2020).

In response to our first research question, we found that religion and spirituality are present in the lives of our informants in a variety of ways, and play a significant role in their meaning making. Some of the informants find their spiritual and social home within traditional religious institutions, such as the Lutheran Church, and find comfort and a sense of belonging through practices, such as worship and reading the gospel. Others have chosen the paths of spiritual seeking that lead them away from these traditional religious practices and toward new expressions of spiritual meaning making, such as meditation or yoga. In this sense, the Finnish retirement migrants in this study confirm the findings from the literature in our theoretical framework about the varied religious and spiritual orientations of the boomer generation.

A significant finding is that for some informants, traditional religious settings, such as church services serve a meaning making function that is more social than it is substantially religious—for them, meeting other Finns and belonging to the community seems to surpass the importance of religious teachings. Yet, it is important to note that despite these seemingly rather functional reasons to stay connected with religious institutions, the sense of social belonging and connectedness they gain from their engagement with it has acquired a spiritual status for many informants. Feeling related to other people and being there for one another in a religious and spiritual context are highly important values in this community, with a great potential for meaning making. Individual spiritual journeys are often interwoven with more traditional religious practices, though for some respondents, their spiritual path has led them to turn away from institutionalized religion permanently.

A finding related to the above is that religion and spirituality turn out to be not so easy to separate in our results. There exists a variety of religious and non-religious forms of spirituality in this population and all of these forms can be relevant factors in meaning making. It is noteworthy to mention that the distinction between religious dwellers and spiritual seekers as laid out in the literature (Wuthnow, 1998; Roof, 1999; Wink, 2003) cannot be clearly drawn in our sample; in a sense, many of our informants appeared to be seeking in some way, and this seeking could occur both within and outside of more traditional religious practices. This is consistent with the idea that the baby boom generation is characterized by multiple religious belongings (Berghuijs, 2017). These results underline the importance of looking beyond dichotomies, such as religious—non-religious or even religious—spiritual when the aim is to understand the role of religious and spiritual phenomena in the lives and meaning making of individuals and communities. The results also emphasize the need to come to a more nuanced understanding of the diversity of experiences related to religious and spiritual meaning-making of older persons—be they in religious or non-religious contexts.

Another important finding to mention is that people exhibited a variety of different motives for including religion and/or spirituality in their meaning-making practices. As indicated before, socially oriented motives, such as seeking connectedness or belonging, caring, and taking responsibility for others, or doing good deeds that benefit individual recipients or the community as a whole, play an important role in meaning making, and are explicitly framed as spiritually inspired motives. On the other hand, there is a group of more individually oriented motives that we propose to unite under the heading of authenticity, which covers the striving for personal growth and spiritual development. While many respondents continue to feel a connection to religious institutions, even if only for its social functions, it is characteristic for most of them that they want to actively create their own spiritual journey. As Atchley puts it, people are “(.) actively creating mosaics of spiritual practice, lifestyles, and communities specifically designed to nurture their vision of the spiritual journey. The spiritual journey is a much different prospect and experience in the evolving dwelling-seeking-practice framework than that experienced in a more static framework that emphasizes authority structures and one-size-fits-all approaches to the spiritual path” (Atchley, 2016, p. 26). This leads us to an interesting direction to answering our second research question in this paper, with regard to how the role of religion and spirituality can enhance our understanding about the relationship between individual and social elements of meaning making. It seems to be the case that whereas individual and social factors are both indispensable in the religious and spiritual meaning making of these retirement migrants, it is vital to them that an authentic and active subject position is maintained always. But contrary to the dominant modern ideal of the authentic, self-sufficient human agent with its problematically atomistic anthropology (Taylor, 1991), for our respondents their authentic subject position is embedded in the social practices of their daily lives, which nourish their individual spirituality and are vital to meaning making.

This study has its limitations. The respondents in all the three datasets are individuals who are at least somewhat intrigued with meaning making and questions related to religion or spirituality. In the future, it would be interesting to examine meaning making with representative material, or in a population not inclined to ponder their lives in any spiritual framework. However, exactly by focusing on persons willing to describe their meaning making, this study offered insights into meaning making in later life as an active process taking place in the social and lived context. These insights should be employed not only in further studies examining meaning in life, but also in the well-being and good life in older age, as this study was one among many highlighting the fundamental importance of meaning making for older persons.

## Data Availability Statement

The raw data supporting the conclusions of this article will be made available by the authors, without undue reservation.

## Ethics Statement

Ethical review and approval was not required for the study on human participants in accordance with the local legislation and institutional requirements. The patients/participants provided their written informed consent to participate in this study.

## Author Contributions

All authors listed have made a substantial, direct and intellectual contribution to the work, and approved it for publication.

## Conflict of Interest

The authors declare that the research was conducted in the absence of any commercial or financial relationships that could be construed as a potential conflict of interest.

## Publisher's Note

All claims expressed in this article are solely those of the authors and do not necessarily represent those of their affiliated organizations, or those of the publisher, the editors and the reviewers. Any product that may be evaluated in this article, or claim that may be made by its manufacturer, is not guaranteed or endorsed by the publisher.

## Appendix A. Interview questions (dataset 2)

1. How did you end up living in the Costa de Sol?
2. How do you spend time here?
3. How 'Finnish' would you say your life is here?
4. What is your relationship to religion and/or spirituality?
5. There is a lot of volunteering here, what's your relationship to that?
6. Quite a few Finns attend religious services here. How is it with you?
7. What do you expect from the future?

## Appendix B. Survey questions (dataset 3)

1. Please write about your religion or spirituality.
2. In what kind of religious or spiritual practices do you engage?
3. Do you believe in God? If not: In what do you believe?
4. What do you think is the meaning of life?
5. Would you say that your religious/spiritual beliefs and your faith in 'God' have changed over the years? How?
6. During the past 6 months, have you participated in an activity, group, or a lecture about good life, personal, or spiritual growth, or religious/spiritual matters?
7. With whom do you discuss questions related to meaning in life or religious/spiritual questions?
8. What does discussing these questions mean to you?
9. What kinds of activities related to these themes would you be interested in?
